# Coordinated regulation of WNT/β-catenin, c-Met, and integrin signalling pathways by miR-193b controls triple negative breast cancer metastatic traits

**DOI:** 10.1186/s12885-021-08955-6

**Published:** 2021-12-04

**Authors:** Chiara Giacomelli, Janine Jung, Astrid Wachter, Susanne Ibing, Rainer Will, Stefan Uhlmann, Heiko Mannsperger, Özgür Sahin, Yosef Yarden, Tim Beißbarth, Ulrike Korf, Cindy Körner, Stefan Wiemann

**Affiliations:** 1 Division of Molecular Genome Analysis, German Cancer Research Center (DKFZ), Heidelberg, Germany; 2 Present address: CRUK Beatson Institute, Bearsden, Glasgow UK; 3 Medical Bioinformatics, University Medical Center Göttingen, Göttingen, Germany; 4 Division of Applied Bioinformatics, German Cancer Research Center (DKFZ), Heidelberg, Germany; 5 Genomics and Proteomics Core Facility, German Cancer Research Center (DKFZ), Heidelberg, Germany; 6 Present address: Department of Drug Discovery and Biomedical Sciences, University of South Carolina, Columbia, SC USA; 7 Department of Biological Regulation, Weizmann Institute of Science, Rehovot, Israel

**Keywords:** Triple negative breast cancer, microRNAs, WNT/β-catenin, c-Met signalling, Integrin signalling

## Abstract

**Background:**

Triple negative breast cancer (TNBC) is the most aggressive subtype of breast cancer (BC). Treatment options for TNBC patients are limited and further insights into disease aetiology are needed to develop better therapeutic approaches. microRNAs’ ability to regulate multiple targets could hold a promising discovery approach to pathways relevant for TNBC aggressiveness. Thus, we address the role of miRNAs in controlling three signalling pathways relevant to the biology of TNBC, and their downstream phenotypes.

**Methods:**

To identify miRNAs regulating WNT/β-catenin, c-Met, and integrin signalling pathways, we performed a high-throughput targeted proteomic approach, investigating the effect of 800 miRNAs on the expression of 62 proteins in the MDA-MB-231 TNBC cell line. We then developed a novel network analysis, Pathway Coregulatory (PC) score, to detect miRNAs regulating these three pathways. Using in vitro assays for cell growth, migration, apoptosis, and stem-cell content, we validated the function of candidate miRNAs. Bioinformatic analyses using BC patients’ datasets were employed to assess expression of miRNAs as well as their pathological relevance in TNBC patients.

**Results:**

We identified six candidate miRNAs coordinately regulating the three signalling pathways. Quantifying cell growth of three TNBC cell lines upon miRNA gain-of-function experiments, we characterised miR-193b as a strong and consistent repressor of proliferation. Importantly, the effects of miR-193b were stronger than chemical inhibition of the individual pathways. We further demonstrated that miR-193b induced apoptosis, repressed migration, and regulated stem-cell markers in MDA-MB-231 cells. Furthermore, miR-193b expression was the lowest in patients classified as TNBC or Basal compared to other subtypes. Gene Set Enrichment Analysis showed that miR-193b expression was significantly associated with reduced activity of WNT/β-catenin and c-Met signalling pathways in TNBC patients.

**Conclusions:**

Integrating miRNA-mediated effects and protein functions on networks, we show that miRNAs predominantly act in a coordinated fashion to activate or repress connected signalling pathways responsible for metastatic traits in TNBC. We further demonstrate that our top candidate, miR-193b, regulates these phenotypes to an extent stronger than individual pathway inhibition, thus emphasizing that its effect on TNBC aggressiveness is mediated by the coordinated repression of these functionally interconnected pathways.

**Supplementary Information:**

The online version contains supplementary material available at 10.1186/s12885-021-08955-6.

## Background

Triple negative breast cancer (TNBC) is a heterogeneous subtype of breast cancer, histologically characterised by absent expression of oestrogen- (ER), progesterone- (PR), or HER2 receptor. Compared to other breast cancer subtypes, TNBC displays the lowest 5-year survival rates, regardless of the stage at diagnosis [1]. Additionally, TNBC patients’ 5-year survival dramatically decreases to 65 and 12.2% if the disease had already spread to regional lymph nodes or at distal sites at the time of diagnosis, respectively [1]. Metastatic recurrence has remained the main cause of cancer-related deaths for all breast cancer patients [2] and thus represents a major challenge for TNBC patients. Indeed, they present the highest percentages in both local and distant recurrences, with metastases more common in brain and lungs [3]. As well, median duration of survival with distant metastasis is the lowest for TNBC (0.5 years) compared to other subtypes (2.2 for LumA, 1.6 LumB, 0.7 Her2+) [4].

The intrinsic heterogeneity of TNBC tumours is a double-edged sword, concomitantly underlying unpredictable differences in response to chemotherapeutic treatments while also presenting itself as potential source of therapeutic vulnerabilities to explore. For the majority of TNBC patients the only viable treatment option is chemotherapy, with responses ranging from pathological complete response (pCR) associated with high rates of survival (12 to 20% of patients), to residual disease after neoadjuvant treatment. Importantly, the latter is associated with significantly worse survival, particularly in the first 3 years (68% vs 98% for patients with pCR) [5]. More recently, four subtypes of TNBC were identified, Basal-Like1 and 2 (BL1 and BL2), Mesenchymal (M), and Luminal Androgen Receptor (LAR). BL2 patients have the lowest probability of reaching a pCR among all TNBC subtypes, and the lowest distant relapse free survival [3]. Thus, TNBC as a heterogeneous disease and the BL2 subtype specifically require deeper biological investigations to fully understand the pathological mechanisms that underlie its clinical aggressiveness, as well as to identify viable novel therapeutic avenues.

The prime function of microRNAs (miRNAs) is to negatively regulate the expression of their target genes at post-transcriptional level by interacting with their 3′ untranslated regions (UTRs). The extent of this regulation has been characterized both at the transcriptomic and proteomic levels, indicating that while regulating a multitude of targets, this happens in a mild fashion. Indeed, various studies have used gain-of-function or loss-of-function miRNA experiments that showed an effect ranging between − 0.3 log2FC [6] and + 0.15 log2FC [7], respectively. In more recent years, the scientific community came to appreciate that miRNAs functionally relevant for specific phenotypes regulate multiple targets within the same signalling cascade [8]. Indeed, a high throughput screening (HTS) at the proteomic level identified miR-193a, miR-124, and miR-147 as regulators of proliferation dependent on their function on the EGFR signalling pathway [9]. Additionally, members of the miR-200 family were characterized to cumulatively affect proteins involved in actin cytoskeleton remodelling, regulating invasion and invadopodia formation [10]. As well, in adult mouse neurons, miR-128 was identified as a decisive regulator of neuronal excitability, due to its ability to control the expression of various ion channels and ERK2 signalling [11]. A comprehensive review has revisited in depth all these phenotypes and network functions of miRNAs showing how they might be additionally integrated in feed-forward and feedback networks, providing insights into the effects that miRNAs have in the context of cancer [8].

Due to their dose-sensitivity, biological pathways require fine-tuned control of the signalling cascade. In these contexts, miRNAs may become pivotal regulators, thanks to their ability to direct the expression of multiple targets [12]. Thus, in this study we aimed to identify miRNAs with a functional relevance in TNBC, mediating a coordinated regulation of signalling pathways. We focused on the WNT/β-catenin, c-Met, and integrin signalling pathways due to their enrichment in the BL2 subtype, which is characterised by worse clinical features [13]. To address the global effects of miRNAs on these pathways, we performed a targeted quantification of proteins upon miRNA gain-of-function in MDA-MB-231 cells, a model of BL2 TNBC. Subsequently, we developed a novel network analysis integrating the effect of miRNAs on proteins’ expression with the function of the same proteins on the pathways of interest, an essential information frequently overlooked in network analysis approaches. We further confirmedmiR-193b as a new strong repressor of all three pathways in TNBC. Of note, its regulatory effects were further validated by negative associations between miRNA expression and pathway activity in gene expression data derived from patients’ datasets.

## Methods

### Cell culture

The human triple negative breast cancer cell lines MDA-MB-231 (Cellosaurus: CVCL_0062) and HCC-1806 (CVCL_1258) were obtained from ATCC (Manassas, VA, USA). SUM-159 (CVCL_5423) cells were a kind gift from Andreas Trumpp (DKFZ, Heidelberg, Germany). All cell lines were authenticated using Multiplex Cell Authentication by Multiplexion (Heidelberg, Germany) as previously described [14]. The SNP profiles matched the expected ones. All cell lines were routinely tested for potential contamination with mycoplasma. MDA-MB-231 cells were cultured in Leibovitz-L15 medium (Gibco, Thermo Fisher Scientific) supplemented with 10% fetal calf serum (Gibco) and 3 g/l of sodium bicarbonate. HCC-1806 cells were cultured in RPMI-1640 (Gibco), supplemented with 10% fetal calf serum (Gibco). SUM-159 cells were cultured in Ham’s F12 (Gibco), supplemented with 5% fetal calf serum (Gibco), 10 mM Hepes (Gibco), 10 μg/ml Hydrocortisone (Sigma, Merck KGaA, Germany), and 5 μg/ml human recombinant Insulin (Sigma). All cell lines were cultured in incubators maintained at 37 °C and 5% CO_2_.

### microRNA gain-of-function experiments

The mimic overexpression screening and cell pellet retrieval were performed as previously described [9]. All additional transient transfections were performed with Lipofectamine2000 (Invitrogen, CA, USA) according to manufacturer’s instructions. miRNA miRIDIAN mimics and respective negative controls, siRNA and respective siRNA negative controls were purchased from Dharmacon (Horizon Discovery, USA) and used at a final concentration of 25 nM. Negative controls in miRNA gain-of-function experiments were miRIDIAN microRNA Mimic Negative Control #1 (CN-001000-01) and #2 (CN-002000-01). For experiments downstream the HTS, the subsequent miRIDIAN microRNA mimics were purchased: miR-193b (C-300764-05), miR-409 (C-300738-05), miR-494 (C-300761-05), miR-92b (C-300872-03). Each of these corresponds in catalogue number and sequence to the mimics present in the library used for the HTS.

### Ectopic activation and inhibition of signalling pathways

The WNT/β-catenin pathway was stimulated with mouse recombinant WNT3a (Peprotech, NJ, USA) at a final concentration of 100 ng/ml. β-catenin transcriptional activity was inhibited by treating cells with iCRT14 (Santa Cruz, CA, USA) at a final concentration of 10 μM. c-Met signalling was stimulated with recombinant human HGF (R&D Systems) at a final concentration of 75 nM, whilst it was inhibited with Capmatinib (Biozol Diagnostica, Eching, Germany) at a final concentration of 2 nM. c-Met and EGFR signalling were concomitantly stimulated with recombinant human EGF (Corning, NY, USA) at a final concentration of 20 nM. Downstream signalling was inhibited with Erlotinib at a final concentration of 5 μM. Recombinant WNT3a, HGF, and EGF were diluted in 0.1% BSA in PBS, which was therefore used as a control in all experiments and is indicated with the “unstimulated” label in respective figures. iCRT-14 and Capmatinib were diluted in DMSO, whilst Erlotinib was diluted in PBS. Thus, the respective vehicle controls (veh. ctrl) were used in the experiments.

### RPPA

RPPA was performed as previously described [15]. Briefly, protein lysates harvested from miRNA-overexpressing MDA-MB-231 cell-pellets were thawed and printed in technical triplicates on nitrocellulose coated glass slides (Oncyte Avid, Grace-Biolabs) using a contact spotter (Aushon BioSystems). Lysates were separated into four groups for spotting. Each of them included appropriate dilution controls for downstream analysis as well as samples transfected with miRNA mimic controls 1 and 2, employed for differential expression analysis (see RPPA HTS data analysis paragraph). Antibody validation for the RPPA screening was performed as previously described [16]. Supplementary Table 1 lists all antibodies used in this study. Unless otherwise noted in Supplementary Table 1, the antibodies were incubated at 1:300 dilution in Blocking buffer. After four washes in Washing buffer, primary antibodies were detected using Alexa Fluor 680 F (ab’)2 fragments of goat anti-mouse immunoglobulin G (IgG) or anti-rabbit IgG (Life Technologies) diluted at 1:8000 in Blocking buffer. Images were acquired at 700 nm wavelength and with 21 μm resolution using an Odyssey scanner (LI-COR, NE, USA). Every nine slides, one was reserved for total protein content analysis by staining with the Fast Green FCF method [17]. Signal intensities were quantified using the GenePix Pro software v.7 (Molecular Devices, CA, USA).

### RPPA HTS data analysis

Signal intensities were processed using the R package RPPanalyzer (v. 1.4.3) [18] for quality control and total protein content normalization. Data quality was assessed by i) checking target specific signals in comparison to their corresponding blank values of the serially diluted control samples and by ii) comparing target measurement signals against blank signals. Spot-wise normalization to the total protein concentration was performed based on the Fast Green FCF method [17]. Potential block effects were removed by shifting the median value of each block to the overall median. The R package ‘limma’ (version 3.26.9) [19] was used to identify miRNAs causing a differential expression of proteins. Within each transfection round, the signal intensities of the miRNA overexpression samples were tested against the two mimic negative control values. Specifically, the comparison was performed between miRNA-transfected samples in two biological replicates against mimic control-transfected samples in two biological replicates of two distinct negative controls. Multiple testing correction was performed with the Benjamini-Hochberg method [20]. For downstream analyses and data plotting, only miRNAs causing at least one statistically significant alteration across the dataset were considered, leading to a final data table and corresponding heatmap of 722 miRNAs by 62 proteins. Statistical significance threshold: q-value ≤0.001. Data analyses were performed in R version 3.2.2.

### Enrichment analyses

Two databases were assessed to retrieve miRNA-target predicted relationships: TargetScanHuman (v.7) [21] and MicroCosm Targets (previously known as miRBase::Targets) [22]. TargetScanHuman database information for conserved and non-conserved targets was individually analysed. Fisher’s exact test was used for enrichment testing, individually addressing downregulated miRNA-target pairs and upregulated miRNA-target pairs. Differential protein expression was considered significant below a threshold of q-value ≤0.001.

### Pathway Coregulatory score analysis

**Fig. 1:**
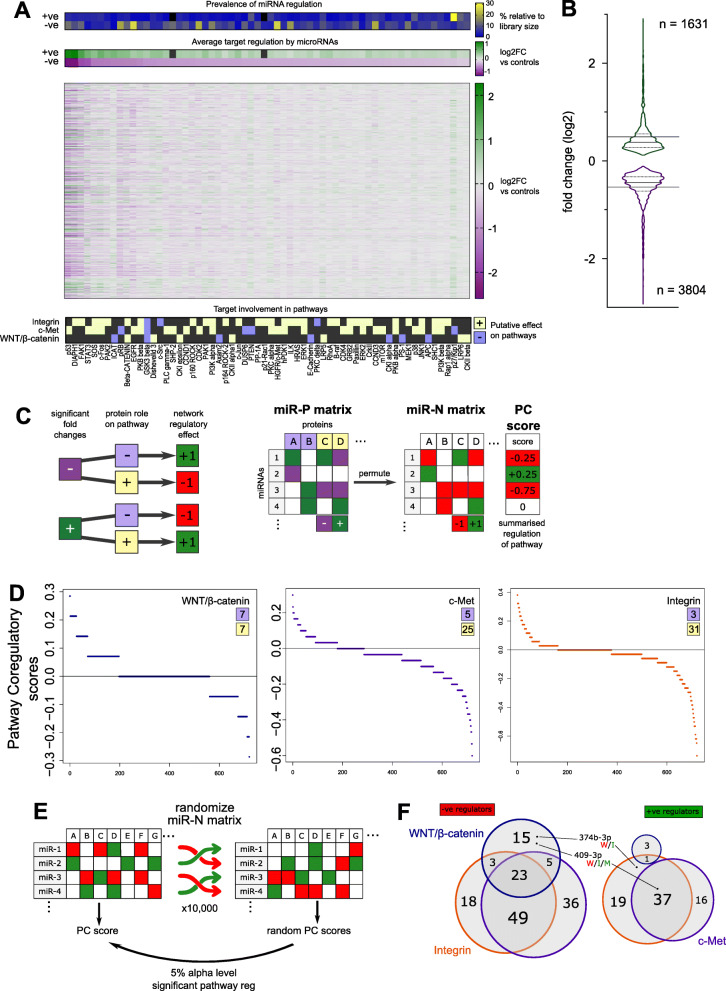
miRNAs coordinately regulate signalling pathways despite mildly regulating individual targets. **A** and **B** – MDA-MB-231 cells were transfected with individual miRNAs from a library of 800 representing the global miRNome. 48 h post-transfection, total protein lysates were harvested and the expression of 62 target proteins was assessed by Reverse Phase Protein Assay (RPPA). After normalization for total protein content, the effect of miRNAs on target proteins was quantified by limma test. *P*-values were corrected for multiple testing with Benjamini-Hochberg method. Tabular results are available in Supplementary Table 2. **A**. Effects of miRNAs on the 62 probed targets are represented with a heatmap of all fold changes compared to negative controls in log2 scale (log2FC). Only miRNAs which caused at least one statistically significant interaction across the entire dataset were plotted, leading to 722 rows. The upper rug represents the prevalence of miRNA regulation for each target, weighted for the library size, separating the number of miRNAs significantly positively (+ve) or negatively (−ve) regulate each target. The second rug represents the average of statistically significant (q-value ≤0.001) regulation of each target, separating positive and negative regulations. The lower rugs represent from which pathway(s) of origin the targets derived from, as well as the putative effect of the target on the pathway. **B**. The regulatory activity of miRNAs is summarised in a violin plot containing all statistically significant (q ≤ 0.001) log2FC in protein expression. Full and dotted lines in the violins respectively represent the medians and the quartiles of the distributions. The horizontal lines in the plot represent the averages. **C**. Principles of PC score computation to transform the effect of a miRNA on a single target protein into a Pathway Coregulatory (PC) effect, integrating the function of the assayed protein on the signalling pathway. miRNA negatively or positively regulate (dark purple and green, respectively) the expression of a target with repressive or activating function (lilac and yellow, respectively). The combination of these two factors identifies the effect on the pathway as positive or negative (bright green or red, respectively). The cumulative effect of a miRNA is then summarized in a PC score classifying each miRNA as activator or repressor of a pathway. **D**. The distributions of computed PC scores for WNT/β-catenin (left), c-Met (middle), and integrin signalling (right). In each graph, numbers indicate the number of putative repressing or activating proteins probed associated to the pathway. **E**. Principles of bootstrapping for statistical testing. The miR-N matrix used to calculate the PC scores was randomized 10,000 times, for each a miRNA-specific random PC score was computed. Then, the experimental PC score was tested against the randomly generated ones. An experimental PC score was considered significant with a 5% alpha level. **F**. Venn diagrams display the number of miRNAs repressing (left) or activating (right) the signalling pathways with a significant effect after randomization test

For pathway analysis, effects on protein levels caused by miRNAs (q-value ≤0.001) were combined with the respective regulatory protein function in the pathway (either activator or repressor) following the rules illustrated in Fig. 1C. Briefly, downregulation of an activator protein in the pathway resulted in a negative pathway effect, and downregulation of a repressor protein in the pathway resulted in a positive pathway effect. The opposite was considered if the miRNA was causing the upregulation of protein expression, Supplementary Table 4 describes the list of targets and the respective biological effect on associated pathway(s). A Pathway oregulation (PC) score was defined for each miRNA as the sum of all measured miRNA-mediated effects on the pathway, weighted by the number of measured proteins in the pathway. Permutation testing was performed to assess the miRNA-wise probability distribution of PC scores by 10,000x resampling the significant miRNA-protein interactions for each protein. PC scores were considered significant based on a 5% FDR. Supplementary file 1 contains the R code in html format employed for the PC score generation and subsequent bootstrap analysis.

### Generation of isogenic recombinant cell lines for WNT pathway reporter assay

MDA-MB-231 were generated as described [23]. Briefly, a mammalian expression vector (pPAR3) containing a Flp recombinase target site N-terminally fused to EGFP under control of an elongation factor 1-alpha (EF1a) promoter and a neomycin selection marker, was stably integrated in the genome of MDA-MB-231 cells. Neomycin resistant and EGFP positive clones were isolated and validated for single-copy integration of the FRT site by Southern blotting. Functionality of the MDA-MB-231-pPAR3 acceptor cell line was verified using Flp-mediated recombination with either a Doxycycline inducible hcRED expression vector for visual testing or a red firefly expression vector for quantitative expression analysis. The validated MDA-MB-231-pPAR3 acceptor cell line served as platform for the generation of isogenic variants.

For generation of MDA-MB-231-pPAR3 WNT/β-catenin-Pathway reporter cell lines, a dual reporter vector containing a promoter-less FRT reporter cassette with TCF/LEF responsive elements followed by a cassette for normalization was flipped into the MDA-MB-231-pPAR3 acceptor cell line by co-transfection with a Flp recombinase expression vector (pOG44 / Invitrogen). The WNT/β-catenin reporter cassette consists of RNA polymerase II transcriptional pause signal from the human hemoglobin subunit alpha 2 gene (*HBA2*) followed by 6 repeats of the TCF/LEF transcriptional response element (AGATCAAAGGGGGTA) joined to a minimal TATA-box promoter and destabilized firefly luciferase reporter (Qiagen, CCS-018 L). The cassette for normalization contains a SV40 promoter driving the renilla luciferase open reading frame, which allows dual measurement of both luciferases. After selection for hygromycin resistance (expression vector) and loss of EGFP expression (positive integration), single colonies were picked and analysed in in the presence of recombinant WNT with dual luciferase assays for WNT / luciferase responsiveness and renilla luciferase expression.

### WNT pathway reporter assay

Three clones of isogenic MDA-MB-231-pPAR3 WNT/β-catenin reporter cell lines were plated at the density of 10,000 cells/well in white flat bottom 96-well plates. The subsequent day, cells were transfected with indicated miRNA mimics or controls. Alternatively, cells were treated with iCRT14. The next day, cells were stimulated with recombinant WNT3a and 18 h later the dual luciferase activities were assayed using a luminometer (Tecan, Männedorf, Switzerland). The median of six technical replicates was used to calculate the ratio over the control. Ratios for three independent clones were averaged and used for statistical testing (two-tailed, one-sample t-test). Statistical testing was performed using GraphPad Prism v9.

### Proliferation and apoptosis assays

Cell lines were plated in black 96-well plates with clear bottom at the indicated densities, based on their respective growth rates: MDA-MB-231 cells at 5000 cells/well, SUM-159 at 1700 cells/well, and HCC-1806 at 2000 cells/well. The next day, cells were transfected with indicated miRNA mimics or negative controls using Lipofectamine 2000. Alternatively, cells were treated with iCRT14, Capmatinib, or Erlotinib. Pathway stimulation was performed concomitantly with chemical inhibition or, for miRNA transfection, 5 h post-transfection upon medium change.

72 h post-treatment, nuclei of cells were stained with Hoechst 33342 (Life Technologies) at a final concentration of 20 μM for 30 min at 37 °C. Subsequently, they were imaged using a Molecular Devices Microscope IXM XLS with 4x S Fluor objective. To assay for apoptotic cells, cells were additionally stained with Propidium Iodide (PI, Life Technologies) at 0.2 ng/mL in addition to Hoechst staining. PI was added 5 min prior to imaging using a Molecular Devices Microscope IXM XLS with 4x S Fluor objective. The percentage of apoptotic cells was assessed by normalizing the PI-positive nuclei to the number of total nuclei (stained with Hoechst).

Image analysis was performed with built-in software. Six technical replicates were performed for each experiment, the mean of the technical replicates was used to calculate the ratio of treatment over control. Ratios of three independent biological replicates were used for statistics (two-tailed tests, for chemical inhibitor experiments: one-sample t-test, for miRNA OE experiments containing two negative control mimics: unpaired t-test). Statistical testing was performed using GraphPad Prism v9.

### Migration assay

MDA-MB-231 cells were plated into clear 6-well plates (Greiner Bio-One) at 400,000 cells/well. The next day, they were either transfected with miR-193b or negative control #2. Alternatively, they were treated with iCRT14 or DMSO control. Two days after transfection, the cells were starved for 18 h with serum-free medium. Subsequently, 200,000 cells were reseeded in serum-free conditions into the upper compartment of 6.5 mm transwell inserts with 5.0 μm pores (Corning), while medium with 10% FCS in the lower compartment was used as chemoattractant. To mimic the inhibitory effect caused by miR-193b overexpression in cells, iCRT14 or DMSO was added both during starvation and upper chamber reseeding, but not in the lower chamber. Overall, reseeding was performed 72 h post-transfection or treatment and migration readout was performed 20 h after reseeding. Inserts were washed with PBS, then cells that had remained within the upper chamber were removed with a cotton swab. Cells that had migrated through the pores were fixed with 4% PFA (prepared from 16% Formaldehyde (w/v), Thermo Fisher Scientific) for 15 min at the lower side of the insert membrane and stained with 20 μM Hoechst 33342 (Life Technologies) for 30 min. Cells were imaged using a Molecular Devices Microscope IXM XLS with 4x S Fluor objective and quantified with built-in software. The mean of three technical replicates was calculated and normalized to a seeding control to account for differences in reseeded cell numbers. Subsequently, a ratio to control condition was calculated and the average for three biological replicates was used for statistics (two-tailed, one-sample t-test). Statistical testing was performed using GraphPad Prism v9.

### FACS analysis of CD24 and CD44 surface expression markers

MDA-MB-231 cells were plated in 6-well plates at a density of 250,000 cells/well. The next day, cells were transfected with miR-193b or mimic negative control #2. Alternatively, cells were treated with iCRT14 or DMSO as control. Four days after transfection, cells were detached with Cell Dissociation Buffer, enzyme-free (GIBCO) and stained for CD-44 and CD-24 surface markers with PE- and APC-conjugated antibodies, respectively (Biolegend). Unstained and isotype controls for APC and PE (Biolegend) were used as controls to gate positive cells. Stained samples were immediately analysed on a FACSCanto II (BD Biosciences). The results were analysed using FACSDiva software (v8, BD Biosciences). Cell percentages from six independent biological replicates with two technical replicates each were used for statistical testing (two-tailed, paired t-test). Statistical testing was performed using GraphPad Prism v9.

### Patient data analysis from TCGA BRCA and METABRIC datasets

miRNA isoform expression quantification analysis from the TCGA cohort was performed using data generated by the TCGA Research Network [24]. The workflow to process the data was based on the British Columbia Genome Sciences Centre miRNA Profiling Pipeline [25]. The harmonized TCGA-BRCA data was downloaded on 7th January 2020 from the Genomic Data Commons Data Portal using the R package ‘TCGAbiolinks’ [26, 27]. The end position of each isomiR feature is exclusive. Thus, the end position annotation was corrected by subtracting 1. For re-annotation of the data, an adaptation of miRBase version 22.1 was applied [28]. All isoforms with the canonical 5′ end of miR-193b-3p but regardless of their 3′ end were summed up and considered as miR-193b [29]. Plate and tumour purity batch effects from sequencing were corrected with the ‘ComBat’ function of the R package ‘sva’ [30].

Normalized miRNA expression data of the METABRIC study [31, 32] was obtained as arbitrary units from the EGA [33, 34]. Data from both discovery and validation sets was merged into a single analysis of miRNA expression in patients. The data was array-based and did not allow discrimination between different microRNA isoforms.

For both datasets, PAM50 classification [35] was directly available in the clinical information, while TNBC status was defined as absence of ER, PR, and Her2 expression at the histological level. Only patients with available PAM50 as well as receptor status were considered, resulting in *n* = 658 patients for TCGA and *n* = 1293 patients for METABRIC. Statistical testing was performed using GraphPad Prism v9 using two-tailed, unpaired t-test.

### Gene set enrichment analysis for TNBC patients

TCGA-BRCA cohort data after batch-correction and isomiR discrimination was used to investigate the correlation between miR-193b expression and the activity of the selected pathways in patients. Batch-corrected expression of miR-193b was used as parameter and batch-corrected mRNA expression data was used as the input file. Gene sets used in this study are: Biocarta_MET and Biocarta_Integrin (from MSigDB), and a manually separated gene list for the positive and negative regulators of WNT (Supplementary Table 6). Spearman correlation coefficients between miR-193b and expressed genes were used as ranking metric and permutation was performed by phenotype. Default parameters of GSEA software [36, 37] were applied.

## Results

### miRNAs mildly regulate expression of proteins belonging to WNT/β-catenin, c-Met and integrin signalling

In a previous study, we addressed miRNAs’ ability to regulate cell cycle dependent on EGFR signalling [9]. Here, we set out to identify miRNAs controlling metastatic traits in TNBC mediated by coordinated regulation of c-Met, integrin, and WNT/β-catenin signalling pathways. We chose to focus on these three pathways as they have been associated specifically with the BL2 subtype of TNBC [13]. Indeed, this subtype is characterised by poor clinical features and thus enriched signalling pathways might qualify as potential therapeutic targets [3]. Importantly, MDA-MB-231, the cell line employed in this high-throughput screening, is specifically a model for the BL2 subtype [13]. To investigate global effects, we employed reverse phase protein array (RPPA), a targeted proteomic approach. We started by selecting and classifying proteins belonging to these three pathways according to the KEGG Network database for c-Met (RTK signalling arm of map 05200), integrin (map 04510), and WNT (canonical WNT arm of map 04310). Proteins originating from cognate mRNAs found expressed below 10 RPKM in an RNA sequencing dataset from MDA-MB-231 cells were excluded from further analyses [38]. We then proceeded to perform antibody validation for specific detection of those proteins whose mRNA was expressed, identifying 62 antibodies appropriate for RPPA (Supplementary Table 1). We performed RPPA on a set of protein lysates derived from a gain-of-function assay where 800 miRNAs had been individually transfected in MDA-MB-231 cells [9]. We quantified by limma test the effect of each miRNA on protein expression, summarising the global effects in a heatmap of log2 fold changes (log2FC) in Fig. 1A (Supplementary Table 2). Considering protein expression downregulation and upregulation separately, the average significant effects of miRNAs overexpression (OE) across all the proteins measured were − 0.54 and + 0.49 log2FC. In both directions we observed maximum effects reaching the absolute values of almost 3 log2FC (Fig. 1B). In the HTS we identified a total of 5435 significant interactions (out of 44,764). Of these, roughly 2/3 were downregulations and 1/3 upregulations (3804 and 1631, respectively). At the individual protein level, we noticed that the adaptor protein SHP-2, and the small GTPase p21-Rac were not significantly upregulated by any miRNA (Fig. 1A, upper heatmap rugs, Supplementary Fig. 1). The remaining 60 targets were significantly upregulated and downregulated by at least one miRNA. However, these effects were not uniform, differing both in number of regulating miRNAs and their extent (Fig. 1A, upper heatmap rugs, Supplementary Fig. 1). Indeed p53, Diap1, FAK1 showed greater log2FC variations compared to other targets, with averages above absolute value of 1 log2FC for both upregulations and downregulations (Supplementary Fig. 1). Focusing on the numbers of regulating miRNAs, low-density lipoprotein receptor-related protein 6 (LRP6) displayed a highly symmetrical regulation with 86 miRNAs upregulating its expression and 59 miRNAs repressing it. Another receptor protein, the EGF receptor (EGFR) was predominantly repressed by miRNAs OE (190 down vs 5 up). Conversely, the cell cycle negative regulator p27/Kip exhibited a predominant upregulation by multiple miRNAs (28 down vs 244 up) (Supplementary Fig. 2A). These strikingly different regulatory patterns prompted us to investigate further the presence of underlying biological features.

miRNA-mediated direct regulation of gene expression occurs predominantly via base-pairing with sequences located in the 3′ UTRs of target mRNAs. Thus, we wondered whether the differences in number of miRNAs regulating a target correlate with the length of the mRNA 3′ UTRs. Specifically, we reasoned that longer 3’UTRs would have a higher chance to harbour miRNA binding sites, thus negatively regulating the expression of the cognate protein. Meanwhile, we expected that upregulation of protein expression upon miRNA OE would more likely be indirect and therefore unrelated to the length of mRNA 3’UTR. To address this, RNA-seq data from MDA-MB-231 cells was exploited to extract cell-specific 3′ UTR lengths, in nucleotides (nt.). There was no correlation between the number of miRNAs significantly regulating the target and the length of the 3′ UTR belonging to the mRNA. Employing an additional cut-off of |0.5| log2FC in defining a significant interaction, we found a positive trend between the number of miRNAs negatively regulating a target and the length of its 3’UTR (Supplementary Fig. 2B, left panel). Unexpectedly, we observed instead a negative trend in correlation for the quantity of miRNAs upregulating protein expression (Supplementary Fig. 2B, right panel). However, both these correlations were not statistically significant. Still, to validate if our RPPA results followed known patterns of protein expression regulation induced by miRNAs, we tested for an enrichment of predicted binding sites (BS) in the 3′ UTR of cognate RNAs [21]. Two independent algorithms were used to generate three separate datasets of predicted miRNA targets: TargetScan Conserved (TSC), TargetScan Non-conserved (TSNC), and microcosm (mC). The enrichment tests were performed independently on the list of miRNA-mediated repressions and on the one of the upregulations. We expected that the increase in protein expression upon miRNA OE would not be mediated by regulations via the 3′ UTR of target mRNAs and thus that there would be no significant enrichment in predicted binding sites. For the downregulating interactions, about half of the targets (32/62) presented a significant enrichment (*p*-value < 0.05) in at least one dataset and roughly one third of the targets (20/62) in two datasets. Among the upregulations, five targets showed a significant enrichment in one dataset while only one, MAPK14 (p38 protein), in two datasets (Supplementary Table 3).

These observations show that the patterns of regulation that we uncovered followed known rules in miRNA-mediated gene expression control at the protein level. Importantly, we identified with high statistical confidence many mild interactions within the three pathways investigated, similarly to what was previously reported [6, 7]. Thus, we concluded that our RPPA dataset reliably quantified significant effects of miRNAs on proteins of interest. Next, we addressed miRNAs’ capability to regulate entire pathways, despite affecting individual targets only mildly.

### miRNAs coordinately control multiple pathways

The fine-tuning patterns identified in the RPPA HTS prompted us to further explore the function of miRNAs as regulators of pathways, shifting the focus from miRNA-protein to a miRNA-network context. We reasoned that the biological effect of a miRNA on a pathway depends on the function of the regulated protein itself. Once this information is integrated in the network, the global effect of a miRNA on a pathway can be summed up based on its regulation of individual targets.

Therefore, we associated each target with the pathways it acts on and assigned a putative positive or negative effect on said pathway based on literature and KEGG pathway descriptors (Fig. 1A lower rug and Supplementary Table 4). Next, we defined that for each miRNA-protein pair, if the miRNA significantly downregulated the expression of a repressor of the pathway, the putative effect on the pathway would be positive. Conversely, if a miRNA downregulates an activator of the pathway, the effect would be negative. The opposite set of rules was applied for miRNAs upregulating their targets (Fig. 1C). For each separate pathway, we transformed the miRNA-to-Protein (miR-P) into miRNA-to-Network (miR-N) effects. These transformed matrices represented the putative effect of a miRNA on the pathway, as mediated by the individual probed target. Next, to describe the cumulative effect of a miRNA on the whole pathway, normalising by the number of targets probed, we generated the Pathway Coregulatory (PC) score (Fig. 1C). The distribution of PC scores is biased by the number of proteins probed per pathway, as well as their putative role. Indeed, for WNT/β-catenin, where we probed 7 activators and 7 repressors, results show a symmetric distribution between repressing and activating miRNAs. On the contrary, for the other two pathways the imbalance caused by probing fewer repressors is shown in a distribution shifted toward repressive miRNA distributions (Fig. 1D).

To robustly identify miRNAs able to regulate the pathways, we tested the PC scores calculated from our experimental data against randomly generated ones (Fig. 1E). miRNAs whose experimental PC score was significantly different than randomly generated one were considered actual pathway regulators (Supplementary Table 5). Among the statistically significant regulating miRNAs, those with a positive experimental PC score were considered activators of a pathway, whilst a negative experimental PC score identified pathway repressors. The results of this analysis show that miRNAs regulating more than one pathway have either a consistent repressive or activating effect on all three pathways (Fig. 1F). The only exceptions to this observed pattern are miR-409-3p, (repressing WNT, while activating integrin and c-Met) and miR-374b-3p (repressing WNT, activating integrin). For each individual pathway, two thirds of the miRNAs significantly negatively regulating it are coregulating in the same direction at least one other pathway, with 23 miRNAs regulating all three pathways. These results are similarly recapitulated for the positive regulations of c-Met and integrin signalling pathways. Interestingly, despite displaying the most symmetric distribution of PC scores, the WNT pathway returned the lowest number of significantly upregulating miRNAs (Fig. 1D and F).

Based on our PC score analysis, we concluded that miRNAs regulating one pathway have the tendency to concordantly regulate other interconnected pathways as well. In turn, this hinted at their ability to affect phenotypes even in the context of mild individual target regulation. Thus, we next sought to validate the capacity of these miRNAs to regulate phenotypes of TNBC by impinging on the selected pathways.

### miRNAs repress WNT/β-catenin and regulate proliferation upon pathway activation

**Fig. 2:**
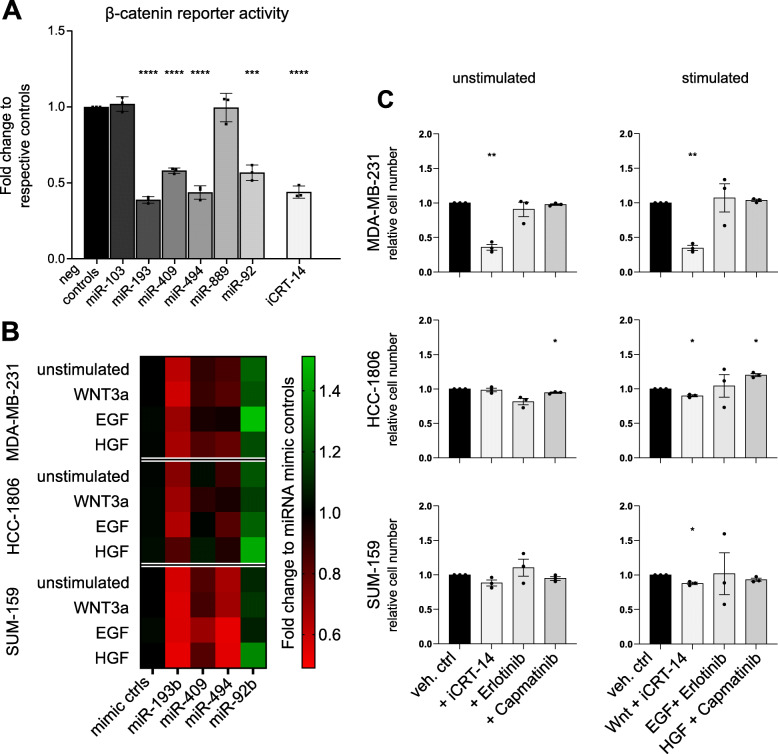
Candidate miRNAs repress pathway activity and pathway-dependent growth. **A**. Stable isogenic Recombinant (SiR) MDA-MB-231 cells were transfected with miRNA mimics or treated with iCRT-14. 30 h later cells were stimulated with recombinant WNT3a. 18 h later, FLuc and RLuc activities were assayed. The effect of miRNAs and iCRT-14 are shown on normalized luciferase activity relative to respective negative controls. Significance was calculated by one-sample, two-tailed t-test on three independent SiR clones. *P*-values *** ≤ 0.0001, *** ≤ 0.001. **B**. MDA-MB-231, SUM-159, and HCC-1806 cells were transfected with miRNA mimics, 5 hours later medium was changed and, where marked, pathways were stimulated. 72 h post-transfection cells growth was evaluated by nuclei counts. The effect of miRNAs was compared to two negative miRNA mimics. Each experiment was repeated in biological triplicate, with six technical replicates each. Growth reduction is represented in red and growth induction in green. Corresponding full bar charts are shown in Supplementary Fig. 3 with statistics. **C**. MDA-MB-231, SUM-159, and HCC-1806 cells were treated with compounds or vehicle controls in combination with pathway stimulations, where marked. 72 h later cell growth was evaluated by nuclei counts. For every condition, the effect of treatments was compared to relative vehicle (DMSO for iCRT-14 and Capmatinib, PBS for Erlotinib; BSA as vehicle control for all stimulations). Being a relative growth, a single control bar is shown in plots. Each experiment was repeated in biological triplicate, with six technical replicates each. Significance was calculated by one-sample, two-tailed t-test. P-values ** ≤ 0.01, * ≤ 0.05. non-significant are not marked

We initially focused on validating miRNAs regulating the WNT/β-catenin signalling network where pathway activity can be quantified based on transcriptional activity driven by TCF/LEF responsive elements. To test if miRNAs identified as repressors are indeed able to reduce the pathway activity, we performed gain-of-function experiment on MDA-MB-231 cells that harbour a stably integrated reporter (i.e., Firefly luciferase) under the control of TCF/LEF responsive elements. Here, six candidate miRNAs were tested: miR-103a-3p, miR-193b-3p, miR-409-3p, miR-494-3p, miR-889-3p, and miR-92b-3p. For all these miRNAs, the 3p arm of the miRNA precursor is the guide miRNA according to miRBase (v22), and as such they will be further indicated without the -3p suffix. As a positive control, we used iCRT-14 (inhibitor of Catenin Responsive Transcription-14), a small molecule that inhibits the interaction between TCF/LEF and β-catenin, thus repressing transcription dependent on the latter [39]. Luciferase activity was evaluated 2 days after transfection with miRNAs and 18 h after stimulation with recombinant WNT3a. Compared to control conditions, miR-889 and miR-103a did not alter reporter gene activity. In contrast, miR-193b, − 409, − 494, and -92b significantly repressed the reporter gene activity by 50% or more (Fig. 2A) indicating efficient suppression of the pathway. A similar level of repression of reporter gene activity was observed upon iCRT-14 treatment. This highlights the high accuracy of the PC score to predict miRNAs regulatory function on the pathway.

Cell cycle induction and proliferation are among the main phenotypes dependent on the activation of the canonical WNT pathway. Thus, we asked whether the four miRNAs identified as repressors of the pathway could regulate cell growth. We investigated the effect of miRNAs and chemical inhibition of the pathway in two additional cell lines representing the same TNBC subtype [3]. MDA-MB-231, SUM-159, and HCC-1806 cells were transfected with miRNA mimics and treated with recombinant WNT3a. Three days after transfection, their growth was compared to the one of controls by counting nuclei as a proxy of proliferation. miR-193b consistently repressed proliferation of all three cell lines, both in WNT-stimulated and unstimulated conditions. miR-494 mildly but significantly reduced cell growth across all cell lines, while miR-409 displayed variable effects depending on the cell line and stimulation conditions. Unexpectedly, miR-92b upregulated proliferation (Fig. 2B and Supplementary Fig. 3 for full bar charts of proliferation experiment). Chemical inhibition of the pathway with iCRT14 strongly repressed proliferation of MDA-MB-231 cells, both in stimulated and unstimulated conditions. However, in the other two cell lines, iCRT-14 treatment had no or little effect (Fig. 2C). Considering the different effects caused by iCRT-14 across cell lines, we hypothesised that the three miRNAs could affect proliferation via different pathways in HCC-1806 and in SUM-159 cells. Hence, we evaluated the effect of these miRNAs on cell growth while stimulating c-Met and its co-interacting partner EGFR with recombinant HGF or EGF, respectively (Fig. 2B). Additionally, we performed chemical inhibition of downstream signalling by treating cells with Capmatinib or Erlotinib (Fig. 2C). The only miRNA retaining growth suppressive capabilities regardless of cell line or stimulation was miR-193b, with the exception of HGF-treated HCC-1806 (Fig. 2B). However, chemical inhibition of c-Met and EGFR signalling pathways did not cause similar effects in any cell line, regardless of stimulation conditions (Fig. 2C and Supplementary Fig. 4).

Concluding, we validated the effect of three miRNAs as repressors of WNT/β-catenin signalling pathway, as well as their ability to suppress cell growth. One of the three miRNAs, miR-193b, displayed a strong phenotype which was not recapitulated by individual pathway suppression with chemical inhibitors. Therefore, we hypothesised that miR-193b functions by coordinate repression of the protein network and proceeded to investigate further phenotypes downstream of WNT/β-catenin and c-Met pathways.

### miR-193b induces apoptosis and represses migration and stem-like features of TNBC cell lines

**Fig. 3:**
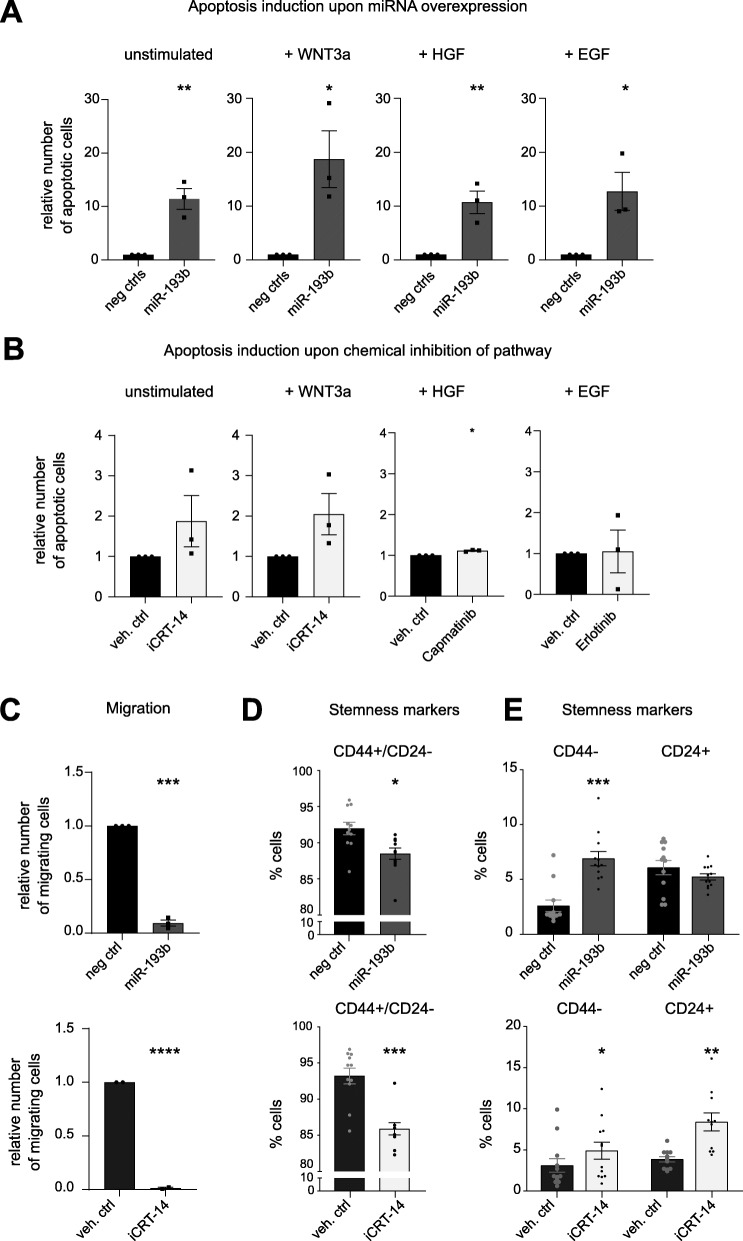
miR-193b regulates apoptosis, migration, and stemness in TNBC cell lines. **A** and **B** – MDA-MB-231 cells were treated and 72 h later apoptosis was evaluated by Propodium Iodide positive nuclei. For each condition, the effect of treatments was quantified relative to respective controls. Unstimulated conditions represent BSA-containing media to the same final concentration present in stimulation conditions, where it was used as carrier protein. **A**The effect of miR-193b was compared to two negative miRNA mimics. **B** The effect of inhibitors was compared to the respective vehicle controls. Each experiment was repeated in biological triplicate, with six technical replicates each. Significance was calculated by two-tailed t-tests (one-sample for chemical inhibitors, unpaired for miRNA OE). P-values *** ≤ 0.001, ** ≤ 0.01, * ≤ 0.05. non-significant are not marked. **C**. Serum-starved MDA-MB-231 cells overexpressing miR-193b or treated with iCRT-14 were seeded in the upper compartment of a transwell system, with serum in the lower chamber as chemoattractant. 20 h later, migration relative to controls was evaluated by nuclei counts. The effect of miR-193b was tested against miRNA mimic negative control #2 (top), and the effect of iCRT-14 against its vehicle, DMSO (bottom). The experiment was repeated in biological triplicate with three technical replicates each. Significance was calculated by one-sample, two-tailed t-test. P-values **** ≤ 0.0001. **D** and **E** – FACS analysis of CD24 and CD44 surface marker expression in MDA-MB-231 cells 96 h post-transfection with miR-193b or iCRT-14 treatment, compared to respective controls. The experiment was repeated in six biological replicates, with two technical replicates each. Significance was calculated by paired, two-tailed t-test. P-values indicated on each graph. P-values *** ≤ 0.001, ** ≤ 0.01, * ≤ 0.05. non-significant are not marked. **D** For each condition, the percentage of cells gated as CD44 positive and CD24 negative (stem-like population) is plotted. **E** For each condition, the percentage of cells gated as CD44 negative (left bars) or CD24 positive (right bars) are plotted

Thus, we next addressed whether miR-193b is capable of regulating apoptosis. As hypothesised, miR-193b increased apoptosis significantly between 10- and 20-fold compared to negative controls. This was evident not only in unstimulated cells, but also when WNT and c-Met pathways were stimulated (Fig. 3A). Conversely, none of the chemical inhibitors induced apoptosis to a similar extent (Fig. 3B). Indeed, only Capmatinib treatment upon HGF stimulation caused a statistically significant increase in apoptosis. However, the drug-mediated effect was only minor, compared to the effect caused by miR-193b (Fig. 3A and B).

The ability of miR-193b to reduce expression of multiple targets within the integrin pathway, including FAK, PAK, and Paxillin, hinted at a function for the miRNA to additionally affect cell motility. Thus, we next tested the effect of miR-193b on migration of MDA-MB-231. Serum-starved cells were transferred in the upper chamber of a transwell system 72 h after miRNA overexpression and were allowed to migrate for 20 h toward serum-containing medium. Migrated cells were quantified by nuclei count and normalised for seeding differences. miR-193b nearly abolished migration toward serum, to a similar extent as cells treated with iCRT-14 (Fig. 3C).

Importantly, WNT pathway is strongly associated with maintenance of stemness in diverse cellular contexts, such as embryonic stem cells, intestinal adult cells, and breast cancer [40, 41]. The stem-like population of cells in breast cancer is characterised by high expression of CD44 and low or no expression of CD24 (CD44+/CD24-). Thus, we tested by Fluorescence Activated Cell Sorting (FACS) the expression of these two surface markers 4 days post-transfection of miR-193b or chemical inhibition of the pathway. Both treatments significantly reduced the stem-like population (CD44+/CD24-) (Fig. 3D). However, analysis of the individual markers showed that miR-193b affected predominantly the expression of CD44, significantly increasing the population of CD44- cells compared to a miRNA mimic control. Oppositely, iCRT-14 treatment did only mildly affect the CD44 expressing population, rather increased the CD24+ cell population (Fig. 3E).

Hence, we demonstrated that miR-193b overexpression in vitro limits not only proliferation, but also additional phenotypes linked with TNBC metastatic traits. Importantly, some of these phenotypes were not recapitulated by individual pathway inhibition, indicating how miR-193b coordinately regulates multiple signalling pathways collectively driving aggressive cancer phenotypes. To further validate the importance of miR-193b in the context of TNBC, we next analysed miRNA expression data derived from BC patients.

### In TNBC patients, miR-193b has lower expression and regulates WNT/β-catenin and c-Met signalling pathways

**Fig. 4:**
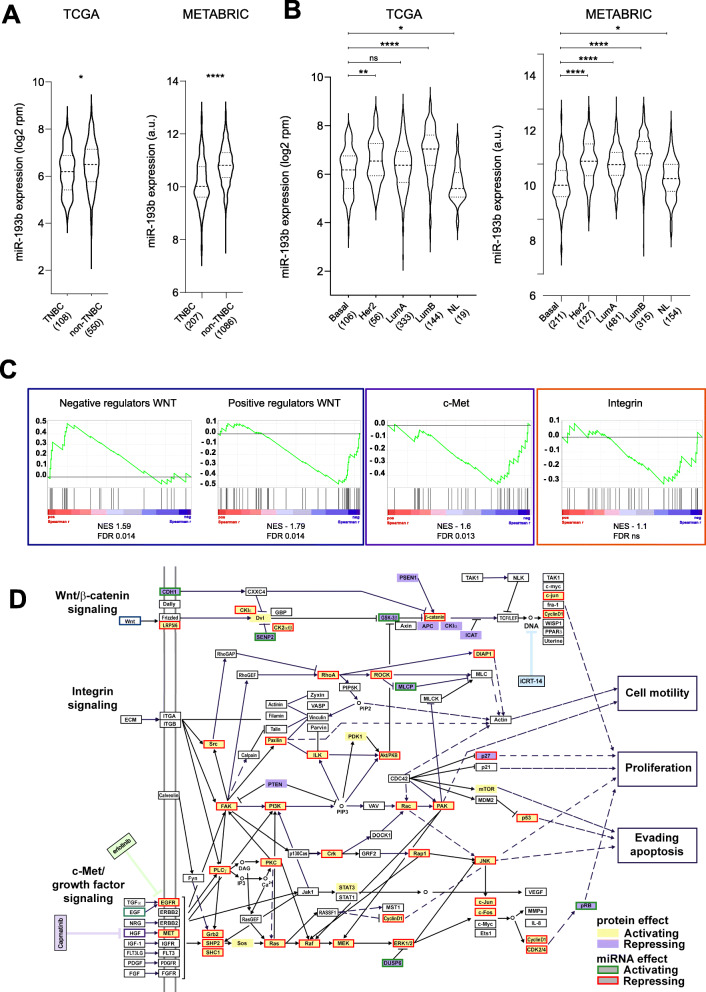
miR-193b expression in BRCA patients is associated with aggressiveness and gene sets of signalling pathways of interest. **A** and **B** – Violin plots of miR-193b expression in two BRCA datasets. Dashed and dotted lines within violins represent the median and quartiles of the distributions. Within each dataset, the number of patients belonging to the TNBC or non-TNBC classification are written in parentheses at the x-axes. Statistical significance was calculated using two-tailed, unpaired t-test. Statistical significance is indicated above comparisons: *p*-values by asterisks **** ≤ 0.0001, *** ≤ 0.001, ** ≤ 0.01, * ≤ 0.05, ns = not significant. **A** miR-193b expression stratifying patients by receptor expression status in TCGA (left) and METABRIC (right) datasets. **B** miR-193b expression in two BRCA datasets stratifying patients by PAM50 classification into Basal, Her2, Luminal A (LumA), and Luminal B (LumB). **C**. Gene Set Enrichment Analysis of gene lists for positive and negative regulators of WNT signalling (blue box), c-Met signalling (purple box), and integrin signalling (orange box). Normalized enrichment scores (NES) and statistical significance by false discovery rates (FDR) are indicated below every signature. **D** Effect of miR-193b on the three signalling pathways, integrated according to their KEGG maps with downstream phenotypes. Target proteins probed in the HTS are shaded in lilac or pale yellow when they are repressors or activators of the pathways, respectively. miR-193b repressive or activating effect on the pathways is represented by a box around the proteins significantly regulated, of red or green colour, respectively. The chemical inhibitors’ activities are highlighted in blue (iCRT-14), purple (Capmatinib), and green (Erlotinib)

Considering miR-193b capabilities in repressing phenotypes associated with aggressiveness in cell models of TNBC, we hypothesised that its expression should be lower in those BC subtypes which are characterised by worse clinical prognosis. Therefore, we analysed miR-193b expression in two independent datasets of breast cancer patients (TCGA BRCA and METABRIC) [25, 31, 32] stratifying them by histological status or by PAM50 classifier. In both datasets miR-193b expression was significantly lower in patients of TNBC subtype (Fig. 4A). Similarly, miR-193b was significantly less expressed in patients classified as Basal by PAM50 signature compared to the other three subtypes (Fig. 4B). Hence, employing two different stratification methods, we demonstrate that miR-193b has a lower expression in the BC subtypes characterised by worse prognosis [1–4].

To consolidate the functional role of miR-193b as a repressor of the pathways of interest, we performed a gene set enrichment analysis (GSEA) on patients’ gene expression data from the TCGA BRCA dataset. First, we selected TNBC patients by their histological status. Then, we correlated miR-193b expression with that of all genes expressed, ranking them from highest to lowest by spearman correlation coefficient. Then, we compiled four lists of genes based on Biocarta (c-Met and integrin signalling), and KEGG (positive and negative regulators of WNT/β-catenin signalling). GSEA indicated that genes encoding for negative regulators of WNT/β-catenin were enriched among the positively correlating genes (Fig. 4C, first plot). Conversely, signatures of positive regulators of WNT (Fig. 4C, second plot) and c-Met signalling (Fig. 4C, third plot) were enriched among the negatively correlated genes. The same trend was observed for the integrin signalling pathway, albeit not reaching significance (Fig. 4C, fourth plot). Hence, these results further support the role of miR-193b as a regulator of the investigated pathways in TNBC patients.

In conclusion, analysing two independent datasets we have shown that miR-193b expression is the lowest in patients diagnosed with the most aggressive subtypes of breast cancer indicating its tumor-suppressive function. Importantly, we confirmed in patient data the correlation between miR-193b and the expression of genes belonging to two of our pathways of interest, WNT/β-catenin and c-Met signalling pathways. Therefore, we concluded that miR-193b is a master regulator of these pathways with pathological relevance in TNBC.

## Discussion

Here we aimed to identify miRNAs which are able to regulate c-Met, integrin, and WNT/β-catenin signalling pathways, thus coordinately controlling phenotypes associated with aggressive traits in TNBC, such as growth, migration, and stem-like features. The effect of miRNAs on the proteins belonging to these three pathways was analysed using a targeted proteomic approach, RPPA. Selecting a very stringent statistical threshold, miRNAs were scored for their putative effect on the selected pathways. The validity of the Pathway Coregulatory (PC) score was demonstrated by a marked downregulation of WNT pathway activity by four out of six miRNAs tested with negative PC scores for WNT. Then, we characterised one of the top-scoring miRNAs, miR-193b, demonstrating its ability to regulate the signalling pathways in TNBC patients’ datasets and in vitro phenotypes dependent on their activity (Fig. 4D).

The proteomics approach we employed addresses the function of miRNAs at the protein rather than mRNA expression level. In the context of ER+ breast cancer, mass spectrometry (MS) coupled with iTRAQ (isobaric tag for relative and absolute quantification) has previously been exploited to identify the targets of miR-193b at the protein level in an unbiased fashion [42]. Gene expression analysis upon miR-193b overexpression showed that only a minority (13%) of the proteins identified had a matched repression of its cognate mRNA [42]. This highlights the importance of considering proteins as they are the functional effectors of signalling pathways and their respectively regulated phenotypes. To overcome the limitations of MS in throughput regarding the number of miRNAs to be investigated, we employed RPPA in this study. We undertook a bootstrap analysis pipeline to quantify effects on pathways and proved that the significant coregulatory effects were greater than random ones, thus overcoming the biases of this targeted approach. While this does not exclude the possibility that the calculated PC scores are still biased due to the selection of probed proteins, the benchmarking analysis of negative regulators of the WNT pathway demonstrates the validity of our setup. Our analysis of multiple pathways showed that miRNAs negatively regulating one tended to have the same function also on neighbouring pathways, thus supporting the concept that miRNAs can effectively regulate complex phenotypes even if their effects are rather mild at the single-target level.

Following our HTS and PC score analysis, we employed a quantitative method to validate the effects of candidate miRNAs. Exploiting a widely employed assay to assess WNT/β-catenin signalling pathway activity, we selected six miRNAs according to two criteria: a) they were identified as repressors of WNT pathway in our PC score analysis, b) they had to be expressed the TCGA dataset, with a minimum average of 10 FPM (fragments per million mapped reads) in TNBC patients. Thus, our approach initially filtered for miRNAs functionally relevant for the regulation of WNT/β-catenin and potentially introduced a bias in candidate selection. However, we support this choice by showing that, with the exception of apoptosis induction, all the phenotypes we investigated are affected also by iCRT-14, a chemical inhibitor of the WNT pathway. Therefore, it is possible that the choice of initially curbing our list of miRNAs of interest with the WNT reporter assay allowed us to identify such a strong miRNA candidate.

The role of the miRNome specifically on the WNT/β-catenin signalling pathway had been previously studied by Anton and colleagues upon transfecting individual miRNAs in HEK293T cells together with a TOP/Flash reporter [43]. The list of miRNAs repressing WNT/β-catenin signalling in our screen was thus compared with the miRNAs assayed in this published screen: four miRNAs were concordantly present in both lists - miR-193b, miR-409, and miR-28 were all described as mild repressors of the reporter gene. On the other hand, miR-181d was characterized as activator of the reporter gene in HEK293Ts. On the contrary, in our PC score analysis all four have been characterised as negative regulators. However, in our experimental validation in MDA-MB-231, only miR-193b and miR-409 downregulated the reporter response, whilst neither miR-28 nor miR-181d could regulate reporter gene expression (unpublished data). Some experimental differences could partially explain the results: e.g. Anton and colleagues transfected miRNA mimics at 40 nM, possibly rendering the screening more prone to off-target and indirect effects when compared to the 25 nM concentration used in our RPPA screening. Nevertheless, biological differences could also explain these divergent results: within a cell system, the presence or absence of specific transcripts, as well as their abundance can deeply affect the role of a miRNA. Therefore, while supporting some of our findings, the screening from Anton and colleagues emphasizes that the effect of miRNAs should be considered in the context of cell and tissue types.

The capacity of miR-193b to concordantly regulate several pathways is of high importance, particularly when compared to the effects we observed with chemical inhibitors of the individual pathways, showing that by targeting at multiple levels, a miRNA exerts a stronger functional output than individual pathway inhibition. The ability of miR-193b to target different pathways and thus coordinately regulate a phenotype is exemplified by the fact that the other two tested cell lines (HCC-1806 and SUM-159) do not respond to inhibition of WNT pathway to the same extent as MDA-MB-231. Thus, their proliferation is not dependent on this pathway. Nevertheless, miR-193b strongly repressed proliferation also in these cell lines indicating alternative mechanisms of action.

Analysis of miR-193b abundance in two independent datasets of BC identified that it is less expressed in more aggressive subtypes, whether classified by histological conditions (TNBC) or molecular characteristics (Basal) (Fig. 4A and B). This indicates that, at least in the context of BC, miR-193b acts as putative tumour suppressor. Nevertheless, its range of expression across all subtypes is wide, indicating that it is not necessarily a driver of aggressiveness.

Our key finding is the capacity of miR-193b to regulate WNT/β-catenin and c-Met pathway in TNBC, both in vitro and in gene expression signatures derived from patients. Previous findings had circumscribed the role of miR-193b as a repressor of individual targets in TNBC, such as its ability to individually downregulate urokinase-type plasminogen activator (uPA) [44], or dimethylarginine dimethylaminohydrolase 1 (DDAH1) [45]. However, possibly pursuing a more physiological avenue, we present the concept that miR-193b exerts its function as tumour suppressor by coordinately regulating entire pathways that are relevant for the acquisition and maintenance of aggressive features. Supported by literature, we recapitulate its effect on growth and migration previously identified [44, 45], and we further characterised its function on apoptosis induction and repression of stem-cell like features, such as the expression of CD24 and CD44 surface markers. Chemical inhibition of WNT pathway via iCRT-14 treatment mimics some of these phenotypes, supporting the idea that they are indeed regulated by WNT pathway. However, based on the discrepancies seen in the response to miRNAs and to chemical inhibition of WNT/β-catenin signalling in the three cell lines assayed, we hypothesize: a) that miRNAs regulate proliferation by affecting multiple pathways, and b) that the proliferation of different cell lines, also riddled by their particular mutation statuses, might depend on different pathways. Thus, we speculate that the same principles apply in vivo as well: miR-193b could have a broad tumour-suppressive function in a heterogeneous patient population thanks to its strong repressive effect on multiple oncogenic pathways.

The WNT/β-catenin signalling pathway has recently gained attention for its effects on TNBC, despite absence of recurrent β-catenin mutations or classical genetic lesions associated with this pathway’s overactivation, such as APC loss in colorectal cancer [46]. Additionally, the pathway was shown to be activated in Basal-like breast cancers (akin to TNBC) where it was associated with worse prognosis [47]. In another study, TNBC patients with a WNT-dependent gene expression signature presented higher rates of lung and brain metastases [48]. At present, the causative role for enhanced activation of WNT/β-catenin signalling has not yet been pinned down to either a common mutation or genomic alteration. It is thus tempting to speculate that its regulation could in part be mediated by miR-193b that we, and others, have found expressed at lower levels in TNBC in patients’ derived gene expression profiles. Additionally, the ability of miR-193b to repress multiple pathways and its reduced expression in TNBC could explain those findings that show how TNBC outcome depends on a combination of deregulated pathways, such as Wnt/β-catenin, c-Met, and CXCL12/CXCR4 [49] [44]. Importantly, a recent study identified a functional link between these three pathways, showing how c-Met and integrin-β1 induce Wnt pathway activation and specific metastatic tissue tropisms [50]. Taken together, this renders re-activation of expression of miR-193b to suppress these interconnected oncogenic pathways an attractive therapeutic approach for TNBC.

## Conclusions

In summary, we developed a new network analysis to unravel miRNAs’ functional relevance on signalling pathways regulating metastatic traits in TNBC. Focusing on WNT/β-catenin, c-Met, and integrin pathways, we identified 23 miRNAs able to repress them in a coordinated fashion. We broadly validated across TNBC cell lines the phenotypic effects of our top candidate, miR-193b-3p. We demonstrated that miR-193b affects phenotypes differently than chemical inhibitors of individual pathways, proving its ability to target them at multiple levels. Ultimately, we showed how TNBC and Basal patients display the lowest miR-193b expression, thus highlighting a potential mechanism that this tumour type employs to activate pro-metastatic signalling pathways.

## Supplementary Information

**Additional file 1.** R code in html format employed for the PC score generation and subsequent bootstrap analysis.

**Additional file 2 Supplementary Fig. 1.** Quantifying effects of miRNAs library overexpression on individual targets. Violin plots of all significant (q ≤ 0.001) positive or negative log2FCs computed by limma testing, for each individual protein assayed. Proteins are ranked by average negative log2FC. p21-Rac, and SHP-2 are displayed at the end of the distribution since they are not significantly upregulated by any miRNA. Within each violin, dashed lines represent the medians and dotted lines represent the quartiles of the distributions. Horizontal red and blue lines represent the averages and medians, respectively, computed from the whole HTS. **Supplementary Fig. 2.** Addressing biological features of transcript regulating protein expression patterns. **A**. Effect of miRNAs on selected targets is represented in three volcano plots. The log2 fold change of protein expression compared to control miRNA mimics is plotted on the x-axes, q-values (in log10) are plotted on the y-axes. Red horizontal lines identify the q-value cutoff used for the downstream analyses (q ≤ 0.001), vertical dotted lines represent for each target the minimum log2FC at which the cutoff was passed. Statistically significant interactions are in purple (downregulations) or green (upregulations). The three targets displayed are Low-density lipoprotein receptor-related protein 6 (LRP6) (left panel), EGF receptor (EGFR) (middle), and p27/Kip (right). **B**. Effect of mRNA 3’UTR sizes on miRNA-mediated regulation. For each target assayed, the length of the 3’UTR of the cognate mRNAs is extracted from MDA-MB-231 sequencing data. Sizes of 3’UTRs on the x-axes (in nucleotides – nt.) are then plotted against the number of miRNAs significantly downregulating (left panel) or upregulating (right panel) the expression of the target proteins by at least an absolute value of 0.5 log2FC. For each distribution, Pearson r is computed and in both cases the *p*-value does not indicate a significant correlation. **Supplementary Fig. 3.** Candidate miRNAs effect on cell growth. Data represented in main Fig. 2B is represented here as a bar chart to allow for individual value and statistically significance inspection. MDA-MB-231, SUM-159, and HCC-1806 cells were transfected with miRNA mimics, 5 hours later medium was changed and, where marked, pathways were stimulated. 72 h post-transfection cells growth was evaluated by nuclei counts. The effect of miRNAs was compared to two negative miRNA mimics. Each experiment was repeated in biological triplicate, with six technical replicates each. Significance was calculated by one-sample, two-tailed t-test. *P*-values **** ≤ 0.0001, *** ≤ 0.001, ** ≤ 0.01, * ≤ 0.05. non-significant are not marked. **Supplementary Fig. 4.** Chemical inhibition of the pathway with reciprocal stimulations. **A – C** – MDA-MB-231, SUM-159, and HCC-1806 cells were treated with compounds or vehicle controls in combination with pathway stimulations, where marked. 72 h later cells growth was evaluated by nuclei counts. For each condition, the effect of treatments was quantified relative to respective controls. The effect of treatments is compared to relative vehicle (DMSO for Capmatinib, PBS for Erlotinib; BSA as stimulation control). Being a relative growth, a single control bar is shown in plots. Each experiment was repeated in biological triplicate, with six technical replicates each. Significance was calculated by one-sample, two-tailed t-test. P-values * ≤ 0.05. non-significant are not marked.

**Additional file 3 Supplementary Table 1.** List of target proteins assayed by RPPA, together with identifiers for the protein (UniProtKB), protein names abbreviations, and identifiers of the cognate transcript genes (HUGO Gene ID, Entrez Gene ID, ENSEMBL Gene ID), as well as information regarding the antibodies used in RPPA (Company, Antibody ID, experimental notes). **Supplementary Table 2.** Results of the limma test on RPPA HTS data. Columns B to BK display corrected *p*-values and columns BN to DW display log2 fold changes computed comparing the effect of miRNAs to the negative controls of the same transfection round. Data is displayed for all the 62 proteins investigated by RPPA. **Supplementary Table 3.** Results of enrichment analysis of predicted interactions in experimental results of RPPA HTS. P-values are marked in green when statistically significant < 0.05. Enrichment is divided into TargetScan conserved (TS Cons), TargetScan non-conserved (TS NC), and microCosm Target (MCT). **Supplementary Table 4.** List of target proteins and respective associated pathway(s). Literature review was done to define the putative effect of the target on the associated pathway(s). **Supplementary Table 5.** After bootstrapping test, miRNAs are considered as repressors of the pathway(s) (− 1, green field) or activators of the pathway(s) (1, red field). The table lists all 722 miRNAs that had at least one statistically significant interaction with one target protein. **Supplementary Table 6.** Gene sets used for GSEA investigating the relationship of miR-193b and the WNT pathway. The positive and negative geneset were generated as subset of the KEGG_WNT_SIGNALING_PATHWAY geneset based on the established function of the encoded proteins within the pathway. **Supplementary Table 7.** Refering to reveiwer 1 concerns: ENCORI database (previously known as StarBase, http://starbase.sysu.edu.cn/index.php) results for miR-193b-3p for targets probed in the RPPA HTS. Narrow and broad sites of binding from the experimental data are shown, together with the number of CLIP experiments reporting the binding (ClipExpNum). RBP column indicates which Argonaute protein have been immunoprecipitated in the experiments listed. The columns of prediction algorithm show whether the discovered binding site is also predicted. The column of pathways associated to our network analysis was added to indicate, of the targets selected, to which pathway they were assigned.Supplementary Table 7 - refering to reveiwer 1 concerns: ENCORI database (previously known as StarBase, http://starbase.sysu.edu.cn/index.php) results for miR-193b-3p for targets probed in the RPPA HTS. Narrow and broad sites of binding from the experimental data are shown, together with the number of CLIP experiments reporting the binding (ClipExpNum). RBP column indicates which Argonaute protein have been immunoprecipitated in the experiments listed. The columns of prediction algorithm show whether the discovered binding site is also predicted. The column of pathways associated to our network analysis was added to indicate, of the targets selected, to which pathway they were assigned.

## Data Availability

The RPPA HTS dataset supporting the conclusions of this article is included within the article in the supplementary table section. The results shown in Fig. 4 are based on data generated by The Cancer Genome Atlas (TCGA) Research Network and by the Molecular Taxonomy of Breast Cancer International Consortium (METABRIC).

## Abbreviations

BC: Breast cancer
TNBC: Triple negative breast cancer
pCR: Pathological Complete Response
miRNA: microRNA
UTR: Untranslated region
HTS: High throughput screening
RPPA: Reverse phase protein array
OE: Overexpression
TSC: TargetScan Conserved
TSNC: TargetScan Non Conserved
mC: Microcosm
miR-P: MiRNA to Protein
miR-N: MiRNA to Network
PC: Pathway Coregulatory
TCGA: The Cancer Genome Atlas
GSEA: Gene Set Enrichment Analysis
MS: Mass spectrometry
