# Supplementary figures and images for: Coordinated regulation of WNT/β-catenin, c-Met, and integrin signalling pathways by miR-193b controls triple negative breast cancer metastatic traits
Source: BMC Cancer. 2021 Dec 4;21:1296. doi: 10.1186/s12885-021-08955-6 (PMC8642942; doi:10.1186/s12885-021-08955-6)

Supplementary figure 1

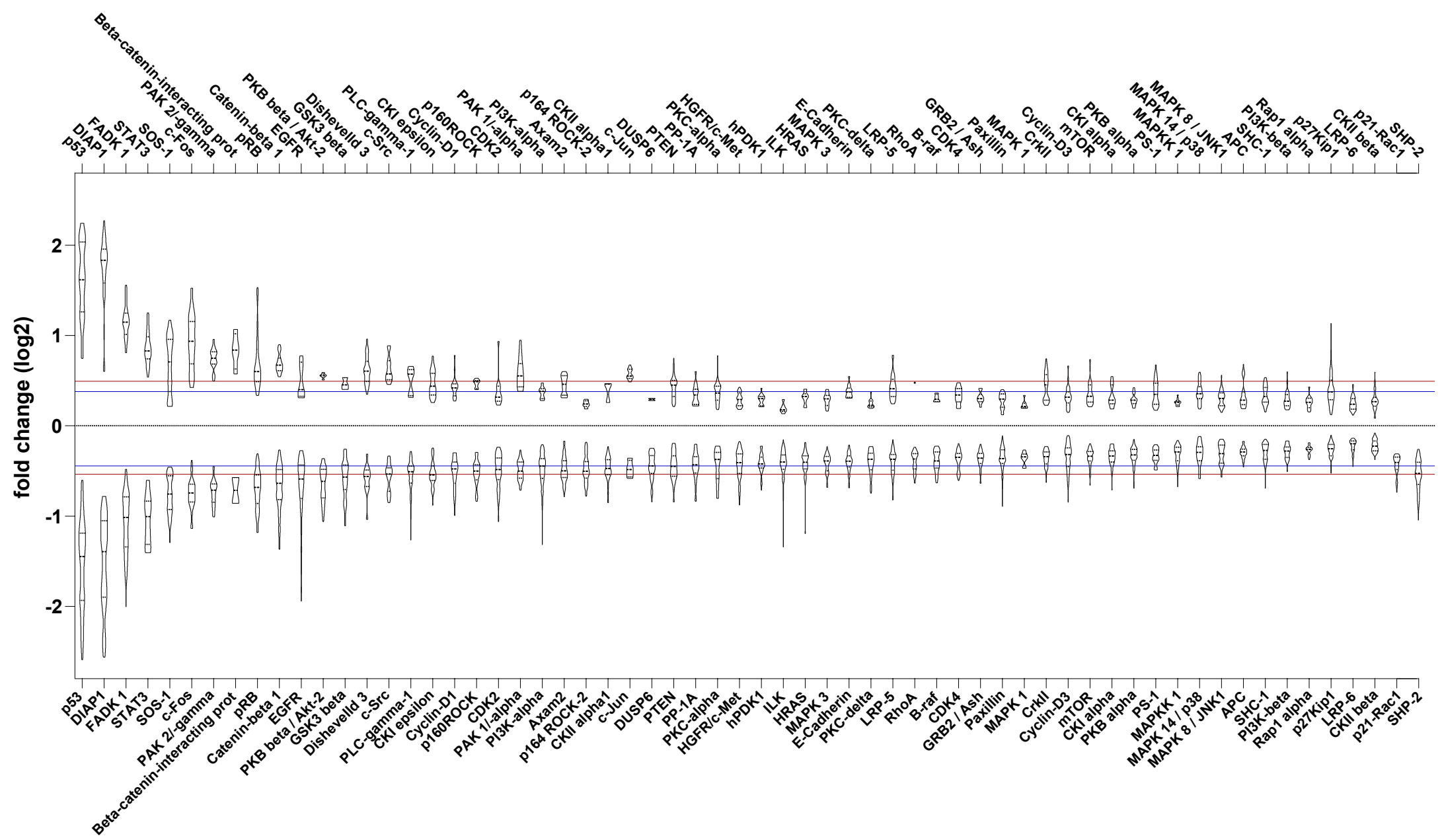

Supplement: Supplementary file 2 — Additional file 2 Supplementary Fig. 1. Quantifying effects of miRNAs library overexpression on individual targets. Violin plots of all significant (q ≤ 0.001) positive or negative log2FCs computed by limma testing, for each individual protein assayed. Proteins are ranked by average negative log2FC. p21-Rac, and SHP-2 are displayed at the end of the distribution since they are not significantly upregulated by any miRNA. Within each violin, dashed lines represent the medians and dotted lines represent the quartiles of the distributions. Horizontal red and blue lines represent the averages and medians, respectively, computed from the whole HTS. Supplementary Fig. 2. Addressing biological features of transcript regulating protein expression patterns. A. Effect of miRNAs on selected targets is represented in three volcano plots. The log2 fold change of protein expression compared to control miRNA mimics is plotted on the x-axes, q-values (in log10) are plotted on the y-axes. Red horizontal lines identify the q-value cutoff used for the downstream analyses (q ≤ 0.001), vertical dotted lines represent for each target the minimum log2FC at which the cutoff was passed. Statistically significant interactions are in purple (downregulations) or green (upregulations). The three targets displayed are Low-density lipoprotein receptor-related protein 6 (LRP6) (left panel), EGF receptor (EGFR) (middle), and p27/Kip (right). B. Effect of mRNA 3’UTR sizes on miRNA-mediated regulation. For each target assayed, the length of the 3’UTR of the cognate mRNAs is extracted from MDA-MB-231 sequencing data. Sizes of 3’UTRs on the x-axes (in nucleotides – nt.) are then plotted against the number of miRNAs significantly downregulating (left panel) or upregulating (right panel) the expression of the target proteins by at least an absolute value of 0.5 log2FC. For each distribution, Pearson r is computed and in both cases the p-value does not indicate a significant correlation. Supplementary Fig. [file 12885_2021_8955_MOESM2_ESM.zip › Supplementary_figR2_1.pdf]

# Supplementary figure 2

A

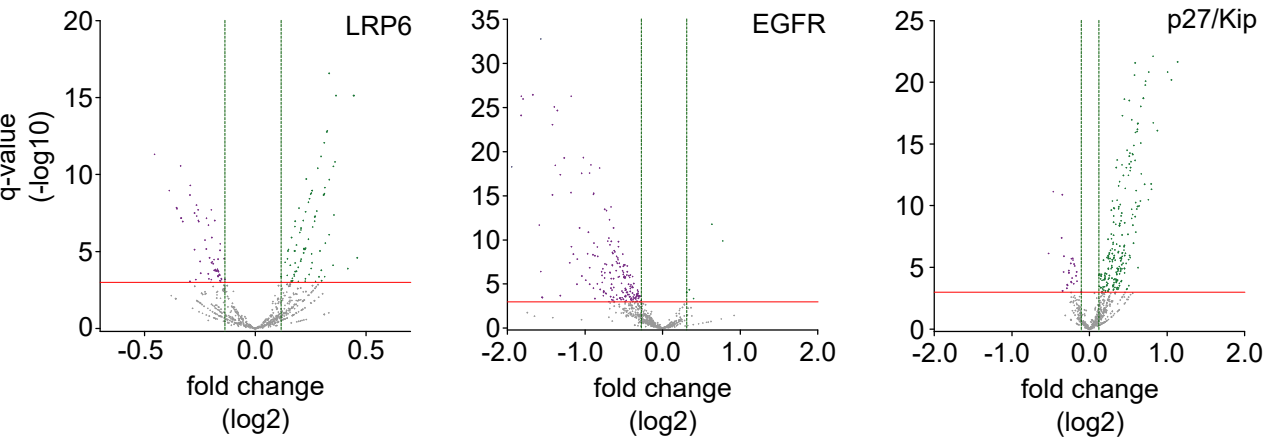

B

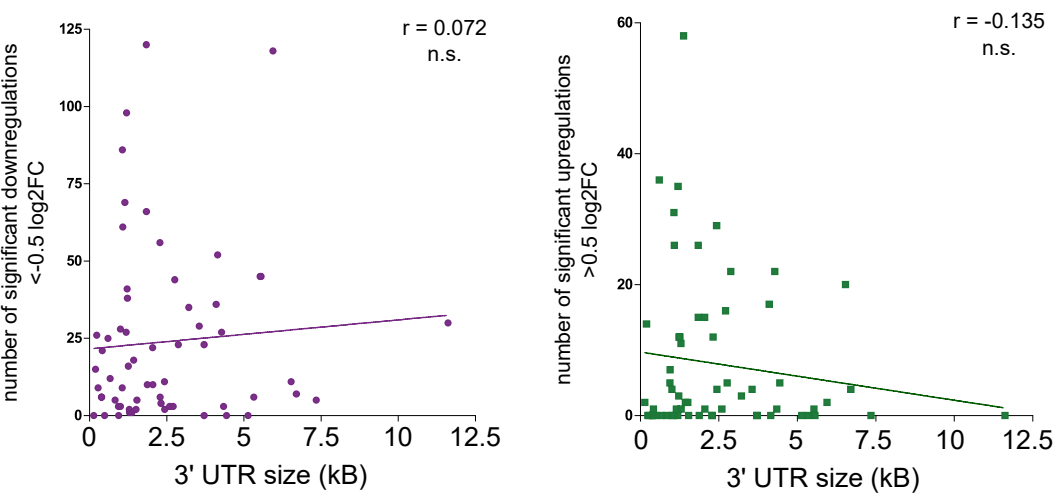

Supplement: Supplementary file 2 — Additional file 2 Supplementary Fig. 1. Quantifying effects of miRNAs library overexpression on individual targets. Violin plots of all significant (q ≤ 0.001) positive or negative log2FCs computed by limma testing, for each individual protein assayed. Proteins are ranked by average negative log2FC. p21-Rac, and SHP-2 are displayed at the end of the distribution since they are not significantly upregulated by any miRNA. Within each violin, dashed lines represent the medians and dotted lines represent the quartiles of the distributions. Horizontal red and blue lines represent the averages and medians, respectively, computed from the whole HTS. Supplementary Fig. 2. Addressing biological features of transcript regulating protein expression patterns. A. Effect of miRNAs on selected targets is represented in three volcano plots. The log2 fold change of protein expression compared to control miRNA mimics is plotted on the x-axes, q-values (in log10) are plotted on the y-axes. Red horizontal lines identify the q-value cutoff used for the downstream analyses (q ≤ 0.001), vertical dotted lines represent for each target the minimum log2FC at which the cutoff was passed. Statistically significant interactions are in purple (downregulations) or green (upregulations). The three targets displayed are Low-density lipoprotein receptor-related protein 6 (LRP6) (left panel), EGF receptor (EGFR) (middle), and p27/Kip (right). B. Effect of mRNA 3’UTR sizes on miRNA-mediated regulation. For each target assayed, the length of the 3’UTR of the cognate mRNAs is extracted from MDA-MB-231 sequencing data. Sizes of 3’UTRs on the x-axes (in nucleotides – nt.) are then plotted against the number of miRNAs significantly downregulating (left panel) or upregulating (right panel) the expression of the target proteins by at least an absolute value of 0.5 log2FC. For each distribution, Pearson r is computed and in both cases the p-value does not indicate a significant correlation. Supplementary Fig. [file 12885_2021_8955_MOESM2_ESM.zip › Supplementary_figR2_2.pdf]

# Supplementary figure 3

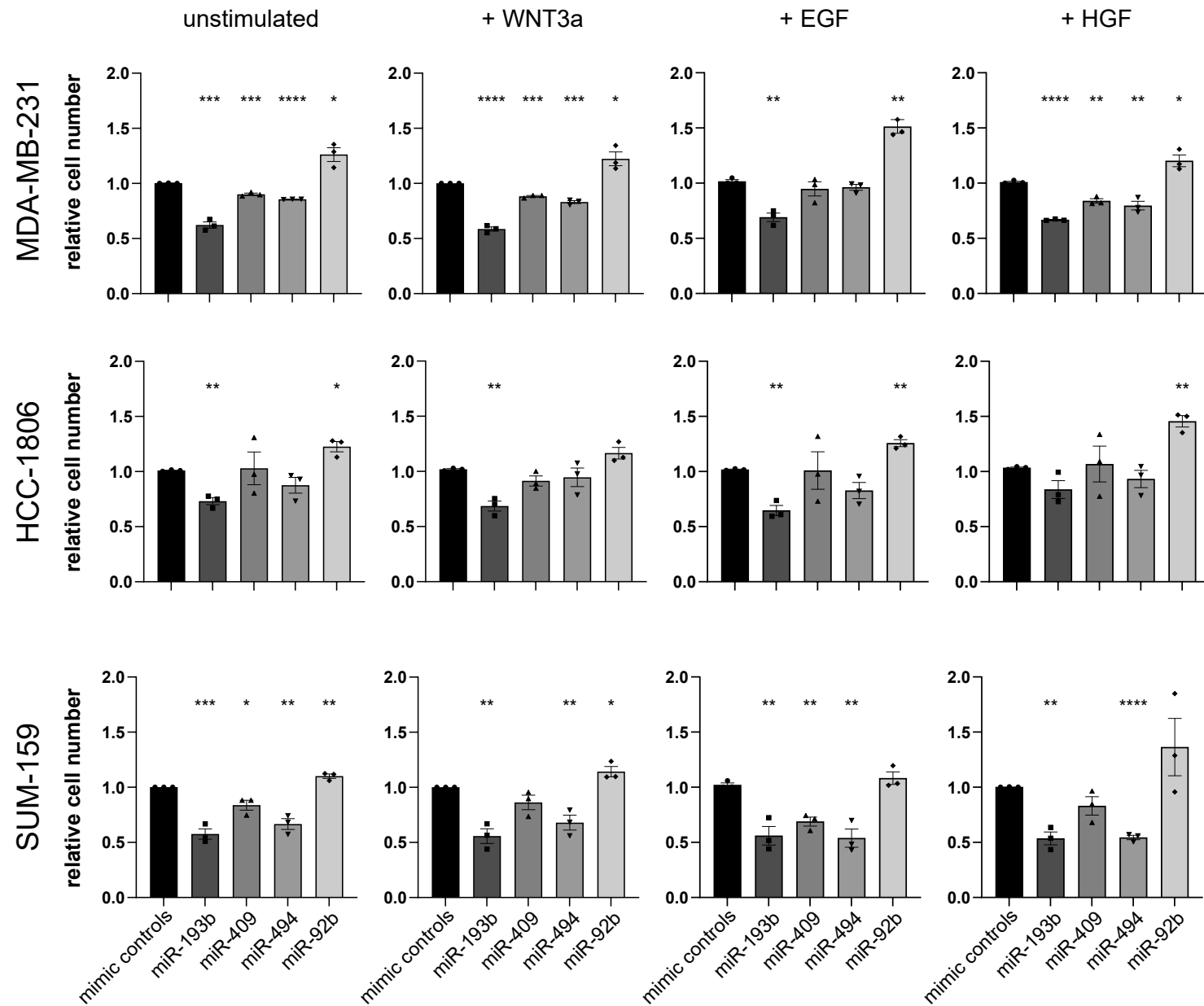

Supplement: Supplementary file 2 — Additional file 2 Supplementary Fig. 1. Quantifying effects of miRNAs library overexpression on individual targets. Violin plots of all significant (q ≤ 0.001) positive or negative log2FCs computed by limma testing, for each individual protein assayed. Proteins are ranked by average negative log2FC. p21-Rac, and SHP-2 are displayed at the end of the distribution since they are not significantly upregulated by any miRNA. Within each violin, dashed lines represent the medians and dotted lines represent the quartiles of the distributions. Horizontal red and blue lines represent the averages and medians, respectively, computed from the whole HTS. Supplementary Fig. 2. Addressing biological features of transcript regulating protein expression patterns. A. Effect of miRNAs on selected targets is represented in three volcano plots. The log2 fold change of protein expression compared to control miRNA mimics is plotted on the x-axes, q-values (in log10) are plotted on the y-axes. Red horizontal lines identify the q-value cutoff used for the downstream analyses (q ≤ 0.001), vertical dotted lines represent for each target the minimum log2FC at which the cutoff was passed. Statistically significant interactions are in purple (downregulations) or green (upregulations). The three targets displayed are Low-density lipoprotein receptor-related protein 6 (LRP6) (left panel), EGF receptor (EGFR) (middle), and p27/Kip (right). B. Effect of mRNA 3’UTR sizes on miRNA-mediated regulation. For each target assayed, the length of the 3’UTR of the cognate mRNAs is extracted from MDA-MB-231 sequencing data. Sizes of 3’UTRs on the x-axes (in nucleotides – nt.) are then plotted against the number of miRNAs significantly downregulating (left panel) or upregulating (right panel) the expression of the target proteins by at least an absolute value of 0.5 log2FC. For each distribution, Pearson r is computed and in both cases the p-value does not indicate a significant correlation. Supplementary Fig. [file 12885_2021_8955_MOESM2_ESM.zip › Supplementary_figR2_3.pdf]

# Supplementary figure 4

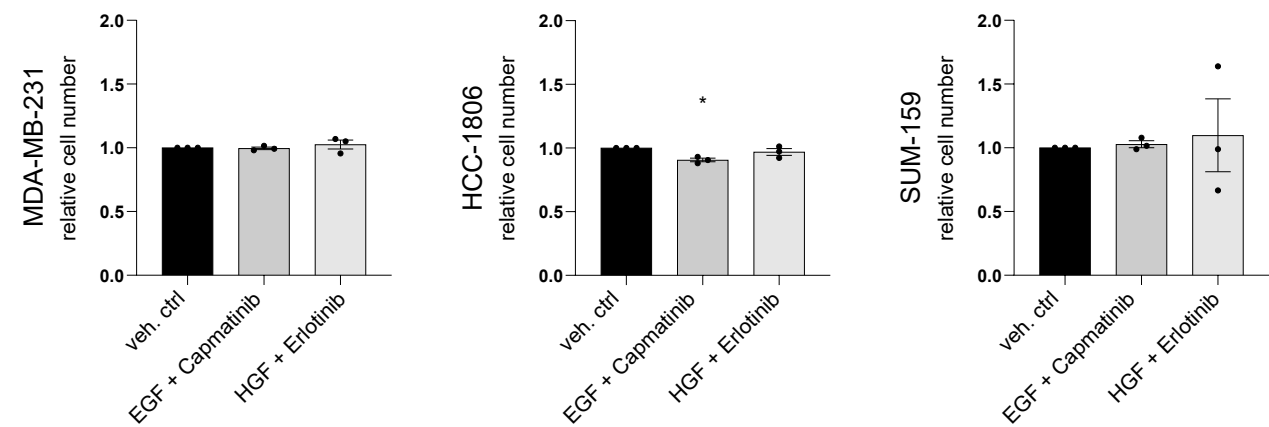

Supplement: Supplementary file 2 — Additional file 2 Supplementary Fig. 1. Quantifying effects of miRNAs library overexpression on individual targets. Violin plots of all significant (q ≤ 0.001) positive or negative log2FCs computed by limma testing, for each individual protein assayed. Proteins are ranked by average negative log2FC. p21-Rac, and SHP-2 are displayed at the end of the distribution since they are not significantly upregulated by any miRNA. Within each violin, dashed lines represent the medians and dotted lines represent the quartiles of the distributions. Horizontal red and blue lines represent the averages and medians, respectively, computed from the whole HTS. Supplementary Fig. 2. Addressing biological features of transcript regulating protein expression patterns. A. Effect of miRNAs on selected targets is represented in three volcano plots. The log2 fold change of protein expression compared to control miRNA mimics is plotted on the x-axes, q-values (in log10) are plotted on the y-axes. Red horizontal lines identify the q-value cutoff used for the downstream analyses (q ≤ 0.001), vertical dotted lines represent for each target the minimum log2FC at which the cutoff was passed. Statistically significant interactions are in purple (downregulations) or green (upregulations). The three targets displayed are Low-density lipoprotein receptor-related protein 6 (LRP6) (left panel), EGF receptor (EGFR) (middle), and p27/Kip (right). B. Effect of mRNA 3’UTR sizes on miRNA-mediated regulation. For each target assayed, the length of the 3’UTR of the cognate mRNAs is extracted from MDA-MB-231 sequencing data. Sizes of 3’UTRs on the x-axes (in nucleotides – nt.) are then plotted against the number of miRNAs significantly downregulating (left panel) or upregulating (right panel) the expression of the target proteins by at least an absolute value of 0.5 log2FC. For each distribution, Pearson r is computed and in both cases the p-value does not indicate a significant correlation. Supplementary Fig. [file 12885_2021_8955_MOESM2_ESM.zip › Supplementary_figR2_4.pdf]
